# 4,15-Dimethyl-7,12-diazo­niatri­cyclo­[10.4.0.0^2,7^]hexa­deca-1(12),2,4,6,13,15-hexa­ene dibromide monohydrate

**DOI:** 10.1107/S2056989020011147

**Published:** 2020-08-18

**Authors:** Edward J. Behrman, Alexandar L. Hansen, Chunhua Yuan, Sean Parkin

**Affiliations:** a Department of Chemistry & Biochemistry, The Ohio State University, 484 W. 12th Avenue, Columbus, Ohio, 43210, USA; b Campus Chemical Instrument Center, The Ohio State University, 496 W. 12th Avenue, Columbus, Ohio, 43210, USA; c Department of Chemistry, University of Kentucky, 505 Rose Street, Lexington, Kentucky, 40506, USA

**Keywords:** viologen, crystal structure, atropisomer, synthesis

## Abstract

The title compound, C_16_H_20_N_2_^2+^·2Br^−^·H_2_O (**1**) is a member of the class of compounds called viologens. Viologens are quaternary salts of di­pyridyls and are especially useful as redox indicators as a result of their large negative one-electron reduction potentials. Compound **1** consists of a dication composed of a pair of 4-methyl­pyridine rings mutually joined at the 2-position, with a dihedral angle between the pyridine rings of 62.35 (4)°. In addition, the rings are tethered *via* the pyridine nitro­gen atoms by a tetra­methyl­ene bridge. Charge balance is provided by a pair of bromide anions, which are hydrogen bonded to a single water mol­ecule [*D*_O⋯Br_ = 3.3670 (15) and 3.3856 (15) Å]. The crystal structure of **1**, details of an improved synthesis, and a full analysis of its NMR spectra are presented.

## Abstract 2

The crystal structure of the viologen 4,4′-dimethyl-2,2′-dipyridyl-*N*,*N*′-tetra­methyl­ene dibromide monohydrate is presented, along with details of an improved synthesis and NMR spectroscopic analysis.

## Chemical context   

The title compound (**1**) is a member of the class of compounds called viologens. Viologens are quaternary salts of di­pyridyls, which have proven useful as redox indicators as a result of their large negative one-electron reduction potentials (Anderson & Patel, 1984 ▸). The herbicides, paraquat, and diquat are viologens. We found that the literature synthesis of 4,4′-dimethyl-2,2′-dipyridyl-*N*,*N*′-tetra­methyl­ene dibromide, *i.e.*, **1** (Spotswood & Tanzer, 1967 ▸) could be improved by a change in the solvent. We report details of our improved synthesis of **1** along with the crystal structure and a full analysis of its NMR spectra.
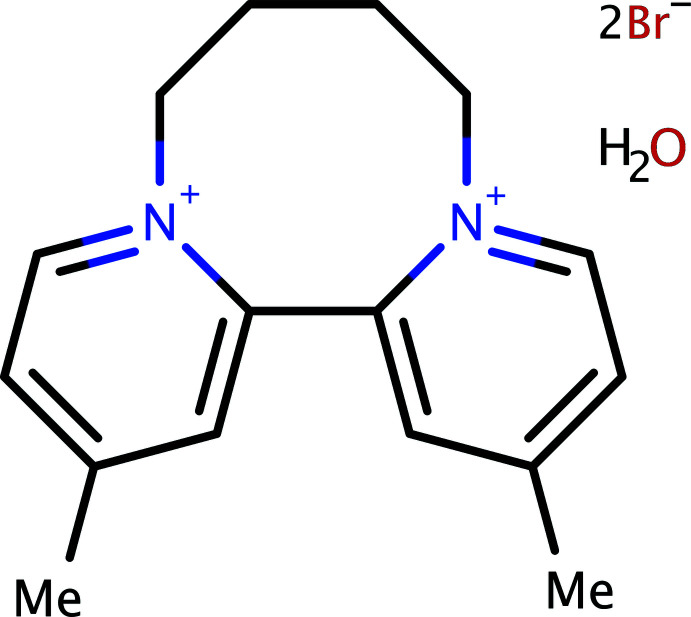


Spotswood & Tanzer (1967 ▸) give general directions for the syntheses of a series of bridged dimethyl 2,2′-dipyridyl salts. Our attempts to make the title compound by their directions failed; only a salt of the starting dipyridyl was recovered. Homer & Tomlinson (1960 ▸) noted that HBr is formed by de­hydro­halogenation of the dibromide. We think that the conditions used by Anderson & Patel (1984 ▸), *i.e.*, refluxing *o*-di­chloro­benzene, b.p. 453 K, produced a good deal of HBr, which protonated the dipyridyl, rendering it unreactive. Carrying out the reaction in refluxing xylene (mixed isomers, b.p. *ca* 413 K) does not produce HBr, but the reaction is slow; after five h, about 50% of the starting dipyridyl was recovered. The quaternization of tertiary amines is known as the Menschutkin reaction (Menschutkin, 1890 ▸). The velocity of this reaction shows a strong dependence on solvent (Abraham & Grellier, 1976 ▸), with about a 65,000-fold increase from hexane to DMSO. The addition of nitro­benzene to the solvent gave satisfactory yields of the product in a reasonable time (see *Synthesis and crystallization* section).

## Structural commentary   

**Figure 1:**
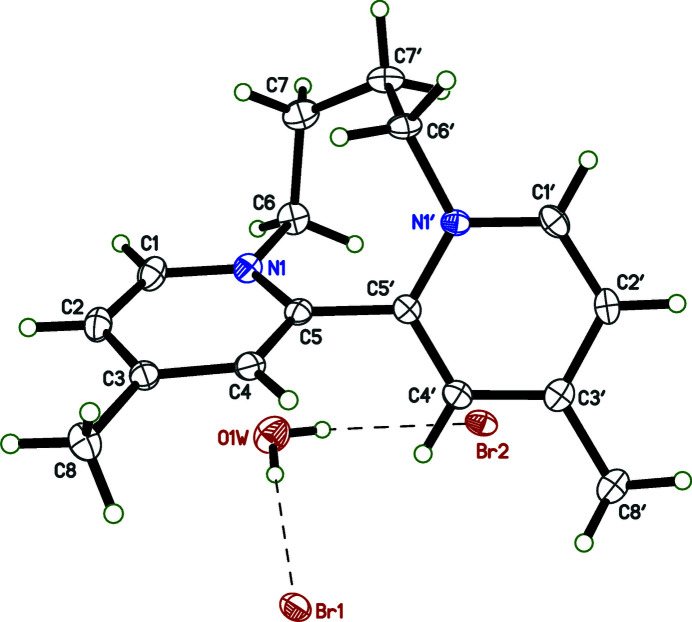
A view of **1** showing the atom-labeling scheme. Displacement ellipsoids are drawn at the 50% probability level. Hydrogen bonds between water and Br^−^ are shown as dashed lines.

The mol­ecular structure of **1** is shown in Fig. 1 ▸. It consists of a dication composed of a pair of 4-methyl­pyridine rings mutually joined at their 2-positions, with a dihedral angle between the pyridine rings of 62.35 (4)°. In addition, the rings are tethered *via* the pyridine nitro­gen atoms by a tetra­methyl­ene bridge. There are no unusual bond lengths or angles. As a result of the two bridges between the pyridine rings, **1** occurs as two optical isomers, and therefore provides an example of atropisomerism (Eliel *et al.*, 1994 ▸; Alkorta *et al.*, 2012 ▸; Mancinelli *et al.*, 2020 ▸). Crystals of **1**, however, were centrosymmetric, with space group *P*2_1_/*n*, and are thus strictly racemic. Charge balance is provided by a pair of bromide anions, which are hydrogen bonded to a single water mol­ecule of crystallization [*D*_O⋯Br_ = 3.3670 (15) and 3.3856 (15) Å] (Table 1 ▸).

## Supra­molecular features   

**Figure 2:**
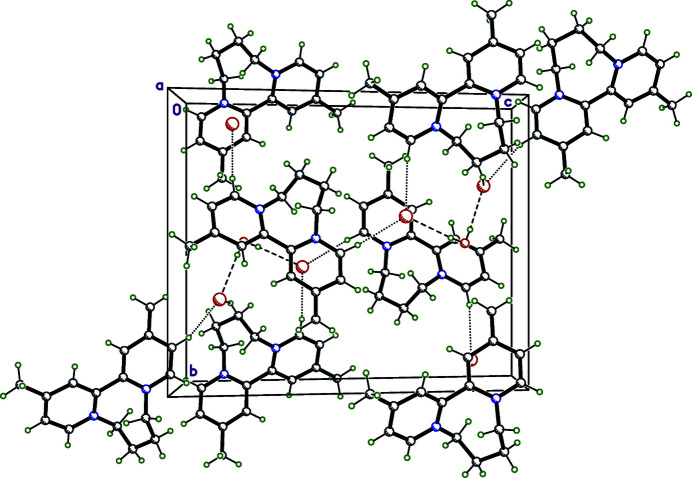
A packing plot of **1** viewed down the crystallographic *a* axis. Hydrogen bonds between water and Br^−^ are shown as dashed lines, while weaker C—H⋯Br inter­actions are shown as dotted lines.

Aside from the hydrogen bonds between the water mol­ecule and bromide anions, the only other notable inter­molecular contacts are inter­actions of type C—H⋯Br (Fig. 2 ▸, Table 1 ▸), with distances that range between 3.5765 (17) and 3.7762 (18) Å for type C_pyrid­yl_⋯Br and 3.6581 (18) to 3.7700 (19) Å for type C_methyl­ene_⋯Br. For comparison, the standard van der Waals radii of C, H, and Br (Bondi, 1964 ▸) are 1.2, 1.7, and 1.85 Å, respectively. The percentages of atom⋯atom contact types between asymmetric units were obtained from Hirshfeld-surface fingerprint plots (Figs. S1 and S2 in the supporting information; Spackman & McKinnon, 2002 ▸; McKinnon *et al.*, 2004 ▸) using *CrystalExplorer 17.5* (Turner *et al.*, 2017 ▸), and are presented in Table 2 ▸.

## Database survey   

The most similar structures to **1** in the Cambridge Structural Database (CSD, V5.41, update of November 2019; Groom *et al.*, 2016 ▸) are BIYTEL, BIYTUB, BIYTOV, BIYVAJ, and BIYTIP (Sanchez *et al.*, 2019 ▸). BIYTEL has a tri­methyl­ene bridge, BIYTUB has a di­methyl­ene bridge, BIYTOV has a tri­methyl­ene bridge but lacks the 4-Me substituents, BIYVAJ has a tri­methyl­ene bridge but 5-Me groups instead of 4-Me, and BIYTIP has a di­methyl­ene bridge but is a methanol solvate. CSD entry TMEPYR (Derry & Hamor, 1970 ▸) contains a tetra­methyl­ene bridge, but lacks 4-Me subsituents. CSD entries DIQUAT (Derry & Hamor, 1969 ▸) and DQUATB (Sullivan & Williams, 1976 ▸), have di­methyl­ene bridges but also lack the 4-Me substituents. Atomic coordinates for TMEPYR, DIQUAT and DQUATB are, however, not present in the CSD. CSD entry PICGAM (Talele *et al.*, 2018 ▸) has a –CH_2_C_6_H_4_CH_2_– linker and is an aceto­nitrile solvate. These crystal structures have Br^−^ anions for charge balance and (unless otherwise stated) include water of crystallization. The tetra­methyl­ene bridge is present in CSD entries HIJGAI (Hofbauer *et al.*, 1996 ▸), YOBWAN (Schmauch *et al.*, 1995 ▸), and YUFCOR (Knoch *et al.*, 1995 ▸), but these crystal structures feature complex organometallic anions rather than bromide and are not hydrates. The dihedral angle between the two pyridine rings in each structure is strongly dependent on the length of the bridging tether. These range between 15.78–19.01° for di­methyl­ene, 49.40–53.96° for tri­methyl­ene, and 63.87–67.15° for tetra­methyl­ene [*cf.* 62.35 (4)° in **1**]. In PICGAM, the dihedral angle is 72.64°, presumably as a result of the increased rigidity of the tether.

## NMR spectroscopic analysis   

**Figure 3:**
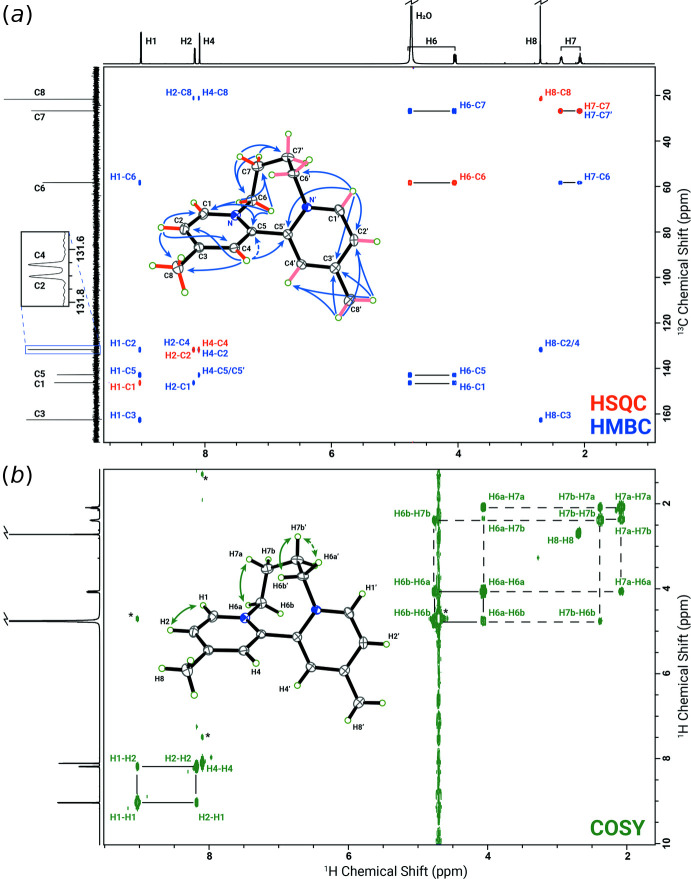
Analysis of 2-D NMR spectra: (*a*) HSQC and HMBC resonance assignments, (*b*) COSY resonance assignments. Peaks marked by an asterisk correspond to water or multiple quantum artifacts. 1-D traces are shown to the left and top of the figure.

The low-field NMR spectrum has been well analyzed by Spotswood & Tanzer (1967 ▸), with whose data we agree. However, the instruments available in 1967 were not able to resolve the bridge protons. Thummel *et al.* (1985 ▸) reported data on the bipyridyl analog, that is, without the 4,4′-methyl groups. Our data closely match theirs (see especially Fig. 3 ▸ of Thummel *et al.*, 1985 ▸), showing an identical complex splitting pattern for the four resolved signals. The protons of the methyl group exchange with deuterons in a base-catalyzed reaction (Zoltewicz & Jacobson, 1978 ▸). Our NMR sample, which also showed exchange, was neutral. Exchange was prevented by adjusting the ‘pH’ to ∼1 with DCl. This exchange with solvent deuterium led to some deuterium couplings with both protons and carbon and hence multiplicities in the NMR spectra, which were initially puzzling. Calder *et al.* (1967 ▸) discuss the effect of the length of the bridging group on the NMR spectra and the mobility of the structures.

There are eight resonance signals in the ^1^H NMR spectrum recorded in D_2_O, including one on the downfield shoulder of the residual water resonance. All but one of the signals are of equal intensity and the one at 2.68 ppm is about three times larger. The ^13^C NMR spectrum shows eight signals (C1–C8), two of which (C2 and C4) are barely separated. Qu­anti­tative measurement using inverse-gated decoupling with a long recycle delay (60 s) shows that the carbon signals are of equal intensity. The 1-D ^13^C DEPT (Distortionless Enhancement by Polarization Transfer) and 2-D multiplicity-edited ^1^H–^13^C HSQC (Heteronuclear Single Quantum Coherence) establish a ratio of 3:2:1 for CH, CH_2_, and CH_3_, respectively. Further analysis of 2-D ^1^H COSY (Correlation Spectroscopy) and 2-D ^1^H-^13^C HMBC (Heteronuclear Multiple-Bond Correlation spectroscopy) spectra led to the NMR assignments summarized in Table 3 ▸. A selective HMBC focusing on the C2/C4 region was recorded for unambiguous assignments of multiple-bond ^1^H–^13^C correlations related to these two carbons. These details together with the 2-D ^1^H–^15^N HMBC, which reveals stronger H2/N9 and H4/N9 cross-peaks than H1/N9, clearly establish a symmetric three-ring mol­ecular structure, as shown in Fig. 3 ▸, in full agreement with the crystal structure (Fig. 1 ▸).

The stereospecific assignment of the methyl­ene protons was achieved by a systematic recording of 1-D selective NOESY (Nuclear Overhauser Effect Spectroscopy) and COSY spectra. A stronger NOE was observed between the proton at 4.73 ppm and H1, and thus this resonance was assigned to H6*A* while the geminal one at 4.03 ppm to H6*B*. The 1-D selective homonuclear decoupling ^1^H NMR spectra led to the extraction of *J*-coupling constants between these methyl­ene protons (Table 3 ▸). A large ^3^ *J* coupling exists between H6*B* and H7*B* (11.3 Hz), followed by a sizable ^3^ *J* coupling between H6*A* and H7*A* (6.1 Hz). As a result of the complexity of the spectra, the ^3^ *J*(H^6*A*^H^7*B*^) and ^3^ *J*(H^6*B*^H^7*A*^) could not be determined, but were estimated to be less than 2 Hz. Also, the 11.1 Hz coupling between H7*A* and H7*B* was tentatively assigned to the geminal coupling rather than the one across the C7–C7′ bond.

All NMR spectra were recorded on a Bruker Ascend 700 MHz spectrometer equipped with a TXO cryoprobe at 298 K. Spectra were indirectly referenced to the deuterium lock frequency, set to 4.7 ppm.

## Synthesis and crystallization   

The starting materials were standard commercial samples of 95-98% purity. 4,4′-Dimethyl-2,2′-dipyridyl (0.92 g, 5 mmol) and 1,4-di­bromo­butane (0.6 mL, 1.08g, 5 mmol) were added to a mixture of 5 mL each of xylene (mixed isomers, b.p. *ca* 413 K) and nitro­benzene (b.p. 483 K). The mixture was refluxed for about 5 h, during which time a heavy precipitate formed. After cooling, the crude material was filtered and washed with acetone to yield 1.1 g of a tan-colored powder. Paper electrophoresis of this material at pH 7.5 showed (*via* UV) a small amount of starting material at *R_p_ ca* zero and product at *R_p_* −2.2 (*R_p_* is movement relative to picric acid). Crystallization from methanol–acetone gave 0.5–0.6 g (ca 50%) of reddish crystals, m.p. 528–530 K [lit. 528–533 K; Spotswood & Tanzer (1967 ▸)], UV_max_(water) 271 nm. IR(Nujol): 3456, 3414, 3372, 1632, 1582, 1566, 1514 1312, 1159, 1032, 853 cm^−1^.

## Refinement   

Crystal data, data collection and structure refinement details are summarized in Table 4 ▸. All hydrogen atoms were found in difference-Fourier maps. Those attached to carbon were subsequently included in the refinement using riding models, with constrained distances set to 0.95 Å (C*sp* ^2^H), 0.98 Å (*R*CH_3_), and 0.99 Å (*R*_2_CH_2_). Water hydrogen coordinates were refined, but subject to a restraint on the O—H distances (*SHELXL* command SADI). *U*_iso_(H) parameters were set to values of either 1.2*U*_eq_ or 1.5*U*_eq_ (*R*CH_3_ only) of the attached atom.

## Supplementary Material

Crystal structure: contains datablock(s) I. DOI: 10.1107/S2056989020011147/ex2036sup1.cif

Structure factors: contains datablock(s) I. DOI: 10.1107/S2056989020011147/ex2036Isup2.hkl

Figure S1. DOI: 10.1107/S2056989020011147/ex2036sup3.tif

Figure S2. DOI: 10.1107/S2056989020011147/ex2036sup4.tif

Supporting information file. DOI: 10.1107/S2056989020011147/ex2036Isup5.cml

CCDC reference: 2023167

Additional supporting information:  crystallographic information; 3D view; checkCIF report

## Figures and Tables

**Table 1:** Hydrogen-bond geometry (Å, °)

*D*—H⋯*A*	*D*—H	H⋯*A*	*D*⋯*A*	*D*—H⋯*A*
C1—H1⋯Br2^i^	0.95	2.68	3.5929 (18)	161
C2—H2⋯Br1^ii^	0.95	2.86	3.7762 (18)	161
C7—H7*B*⋯Br1^i^	0.99	2.96	3.7700 (19)	139
C1′—H1′⋯Br2^iii^	0.95	2.64	3.5765 (17)	170
C2′—H2′⋯Br1^iv^	0.95	2.82	3.6285 (17)	143
C4′—H4′⋯Br1	0.95	2.74	3.6735 (17)	167
C7′—H7*B*′⋯Br1^v^	0.99	3.04	3.6581 (18)	122
O1*W*—H1*W*⋯Br1	0.81 (2)	2.58 (2)	3.3856 (15)	177 (3)
O1*W*—H2*W*⋯Br2	0.81 (2)	2.56 (2)	3.3670 (15)	175 (3)

Symmetry codes: (i) 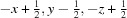; (ii) 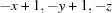; (iii) 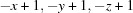; (iv) 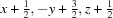; (v) 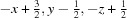.

**Table 2:** Percentage of atom⋯atom contacts between asymmetric units in **1**

H⋯H	57.0
H⋯Br	26.2
H⋯C	9.0
H⋯O	4.7
C⋯Br	1.7
N⋯Br	1.1
C⋯C	0.4
N⋯N	0.0
O⋯O	0.0
Br⋯Br	0.0

Contact percentages were derived from Hirshfeld-surface fingerprint plots (Spackman & McKinnon, 2002 ▸; McKinnon *et al.*, 2004 ▸) using *CrystalExplorer 17.5* (Turner *et al.*, 2017 ▸). Reciprocal contacts are included in the totals. The sum of all percentages in the table is 100.1% due to accumulation of rounding errors.

**Table 3:** ^1^H and ^13^C NMR spectroscopic data for **1** recorded in D_2_O at 298K

Assignments	^13^C (ppm)	^1^H (ppm)	Couplings (Hz)
C^1^/H^1^	146.25	8.99	^3^ *J*(H^1^H^2^) 6.4
C^2^/H^2^	131.70	8.14	^4^ *J*(H^2^H^4^) 1.4
C^3^	162.63		
C^4^/H^4^	131.66	8.07	
C^5^	142.78		
C^6^/H^6*A*^,H^6*B*^	58.26	H^6*A*,6*B*^ 4.73, 4.03	^2^ *J*(H^6*a*^H^6*B*^) 14.5, ^3^ *J*(H^6*A*^H^7*A*^) 6.1, ^3^ *J*(H^6*B*^H^7*B*^) 11.3
C^7^/H^7*A*^,H^7*B*^	26.72	H^7*A*,7*B*^ 2.35, 2.05	^2^ *J*(H^7*A*^H^7*B*^) 11.1
C^8^/H^8^	21.62	2.68	
N^9^	208.5		

The errors were estimated to be ±0.02ppm, ± 0.002ppm, and ±0.3Hz, respectively, for the ^13^C chemical shifts, ^1^H chemical shifts, and *J* coupling constants.

**Table 4:** Experimental details

Crystal data	
Chemical formula	C_16_H_20_N_2_^2+^·2(Br^−^)·H_2_O
*M*_r_	418.17
Crystal system, space group	Monoclinic, *P*2_1_/*n*
Temperature (K)	90
*a*, *b*, *c* (Å)	7.6402 (2), 13.7578 (3), 16.7691 (3)
β (°)	101.162 (1)
*V* (Å^3^)	1729.30 (7)
*Z*	4
Radiation type	Mo *K*α
μ (mm^−1^)	4.69
Crystal size (mm)	0.16 × 0.12 × 0.07

Data collection	
Diffractometer	Bruker D8 Venture dual source
Absorption correction	Multi-scan (*SADABS*; Krause *et al.*, 2015 ▸)
*T*_min_, *T*_max_	0.562, 0.746
No. of measured, independent and observed [*I* > 2σ(*I*)] reflections	26025, 3959, 3527
*R*_int_	0.029
(sin θ/λ)_max_ (Å^−1^)	0.650

Refinement	
*R*[*F* ^2^ > 2σ(*F* ^2^)], *wR*(*F* ^2^), *S*	0.020, 0.043, 1.06
No. of reflections	3959
No. of parameters	198
No. of restraints	1
H-atom treatment	H atoms treated by a mixture of independent and constrained refinement
Δρ_max_, Δρ_min_ (e Å^−3^)	0.38, −0.38

Computer programs: *APEX3* (Bruker, 2016 ▸), *SHELXT* (Sheldrick, 2015*a* ▸), *SHELXL2018/3* (Sheldrick, 2015*b* ▸), *XP* in *SHELXTL* (Sheldrick, 2008 ▸), *SHELX* (Sheldrick, 2008 ▸), *CIFFIX* (Parkin, 2013 ▸), and *publCIF* (Westrip, 2010 ▸).

## supplementary crystallographic information

### Crystal data

**Table:**

C_16_H_20_N_2_^2+^·2(Br^−^)·H_2_O	*F*(000) = 840
*M**_r_* = 418.17	*D*_x_ = 1.606 Mg m^−^^3^
Monoclinic, *P*2_1_/*n*	Mo *K*α radiation, λ = 0.71073 Å
*a* = 7.6402 (2) Å	Cell parameters from 9914 reflections
*b* = 13.7578 (3) Å	θ = 2.8–27.5°
*c* = 16.7691 (3) Å	µ = 4.69 mm^−^^1^
β = 101.162 (1)°	*T* = 90 K
*V* = 1729.30 (7) Å^3^	Irregular shard, pink
*Z* = 4	0.16 × 0.12 × 0.07 mm

### Data collection

**Table:**

Bruker D8 Venture dual source diffractometer	3959 independent reflections
Radiation source: microsource	3527 reflections with *I* > 2σ(*I*)
Detector resolution: 7.41 pixels mm^-1^	*R*_int_ = 0.029
φ and ω scans	θ_max_ = 27.5°, θ_min_ = 2.5°
Absorption correction: multi-scan (*SADABS*; Krause *et al.*, 2015)	*h* = −9→9
*T*_min_ = 0.562, *T*_max_ = 0.746	*k* = −17→17
26025 measured reflections	*l* = −21→21

### Refinement

**Table:**

Refinement on *F*^2^	Primary atom site location: structure-invariant direct methods
Least-squares matrix: full	Secondary atom site location: difference Fourier map
*R*[*F*^2^ > 2σ(*F*^2^)] = 0.020	Hydrogen site location: mixed
*wR*(*F*^2^) = 0.043	H atoms treated by a mixture of independent and constrained refinement
*S* = 1.06	*w* = 1/[σ^2^(*F*_o_^2^) + (0.0121*P*)^2^ + 1.4994*P*] where *P* = (*F*_o_^2^ + 2*F*_c_^2^)/3
3959 reflections	(Δ/σ)_max_ = 0.002
198 parameters	Δρ_max_ = 0.38 e Å^−^^3^
1 restraint	Δρ_min_ = −0.38 e Å^−^^3^

### Special details

**Table:**

Experimental. The crystal was mounted using polyisobutene oil on the tip of a fine glass fibre, which was fastened in a copper mounting pin with electrical solder. It was placed directly into the cold gas stream of a liquid-nitrogen based cryostat (Hope, 1994; Parkin & Hope, 1998). Diffraction data were collected with the crystal at 90K, which is standard practice in this laboratory for the majority of flash-cooled crystals.
Geometry. All s.u.s (except the s.u. in the dihedral angle between two l.s. planes) are estimated using the full covariance matrix. The cell s.u.s are taken into account individually in the estimation of s.u.s in distances, angles and torsion angles; correlations between s.u.s in cell parameters are only used when they are defined by crystal symmetry. An approximate (isotropic) treatment of cell s.u.s is used for estimating s.u.s involving l.s. planes.
Refinement. Refinement progress was checked using *Platon* (Spek, 2009) and by an *R*-tensor (Parkin, 2000). The final model was further checked with the IUCr utility *checkCIF*.

### Fractional atomic coordinates and isotropic or equivalent isotropic displacement parameters (Å^2^)

**Table:**

	*x*	*y*	*z*	*U*_iso_*/*U*_eq_	
Br1	0.38758 (2)	0.69802 (2)	0.11426 (2)	0.01809 (5)	
Br2	0.09621 (2)	0.58825 (2)	0.34885 (2)	0.01872 (5)	
N1	0.54040 (19)	0.38619 (10)	0.23392 (9)	0.0157 (3)	
C1	0.5310 (2)	0.34004 (13)	0.16231 (11)	0.0193 (4)	
H1	0.470042	0.279632	0.153665	0.023*	
C2	0.6074 (2)	0.37819 (13)	0.10161 (11)	0.0190 (4)	
H2	0.598713	0.344145	0.051717	0.023*	
C3	0.6973 (2)	0.46646 (13)	0.1128 (1)	0.0164 (3)	
C4	0.7082 (2)	0.51161 (13)	0.18795 (10)	0.0159 (3)	
H4	0.771186	0.571265	0.198231	0.019*	
C5	0.6300 (2)	0.47175 (12)	0.24753 (10)	0.0140 (3)	
C6	0.4467 (2)	0.34092 (13)	0.29478 (11)	0.0188 (4)	
H6A	0.337285	0.307919	0.266189	0.023*	
H6B	0.410854	0.392247	0.329762	0.023*	
C7	0.5666 (2)	0.26732 (13)	0.34768 (12)	0.0215 (4)	
H7B	0.489315	0.219545	0.368381	0.026*	
H7A	0.635324	0.231572	0.312750	0.026*	
C8	0.7795 (3)	0.51217 (14)	0.04805 (11)	0.0232 (4)	
H8A	0.750594	0.473388	−0.001783	0.035*	
H8B	0.909326	0.514930	0.066178	0.035*	
H8C	0.732611	0.578163	0.037298	0.035*	
N1'	0.72247 (18)	0.4864 (1)	0.39609 (8)	0.0136 (3)	
C1'	0.7337 (2)	0.53707 (13)	0.46545 (10)	0.0172 (4)	
H1'	0.792782	0.508916	0.515149	0.021*	
C2'	0.6619 (2)	0.62861 (13)	0.46625 (10)	0.0170 (3)	
H2'	0.673762	0.663389	0.515955	0.020*	
C3'	0.5719 (2)	0.67031 (13)	0.39448 (11)	0.0161 (3)	
C4'	0.5608 (2)	0.61627 (12)	0.32318 (10)	0.0154 (3)	
H4'	0.500590	0.642672	0.272967	0.018*	
C5'	0.6360 (2)	0.52515 (12)	0.32479 (10)	0.0135 (3)	
C6'	0.8162 (2)	0.39082 (12)	0.40077 (11)	0.0169 (4)	
H6A'	0.925433	0.393784	0.443492	0.020*	
H6B'	0.852567	0.377516	0.348290	0.020*	
C7'	0.6976 (2)	0.30844 (13)	0.41992 (11)	0.0198 (4)	
H7A'	0.629120	0.332138	0.460449	0.024*	
H7B'	0.775237	0.254877	0.445511	0.024*	
C8'	0.4859 (2)	0.76805 (13)	0.39343 (12)	0.0210 (4)	
H8A'	0.500384	0.803271	0.344334	0.031*	
H8B'	0.542276	0.804908	0.441597	0.031*	
H8C'	0.358509	0.760037	0.393689	0.031*	
O1W	0.1964 (2)	0.49717 (11)	0.17678 (9)	0.0297 (3)	
H1W	0.240 (3)	0.5445 (16)	0.1598 (16)	0.044*	
H2W	0.168 (3)	0.5155 (19)	0.2183 (13)	0.044*	

### Atomic displacement parameters (Å^2^)

**Table:**

	*U*^11^	*U*^22^	*U*^33^	*U*^12^	*U*^13^	*U*^23^
Br1	0.01962 (9)	0.02036 (9)	0.01455 (8)	0.00295 (7)	0.00397 (6)	0.00357 (7)
Br2	0.01603 (9)	0.01993 (9)	0.01935 (9)	−0.00218 (7)	0.00129 (6)	0.00533 (7)
N1	0.0134 (7)	0.0157 (7)	0.0178 (7)	−0.0010 (6)	0.0022 (6)	−0.0013 (6)
C1	0.0155 (8)	0.0185 (9)	0.0229 (9)	−0.0014 (7)	0.0012 (7)	−0.0065 (7)
C2	0.0166 (8)	0.0215 (9)	0.0181 (8)	0.0020 (7)	0.0012 (7)	−0.0056 (7)
C3	0.0146 (8)	0.0189 (9)	0.0153 (8)	0.0046 (7)	0.0020 (6)	0.0001 (7)
C4	0.0158 (8)	0.0140 (8)	0.0173 (8)	0.0006 (6)	0.0017 (6)	0.0005 (7)
C5	0.0110 (7)	0.0144 (8)	0.0157 (8)	0.0020 (6)	0.0004 (6)	−0.0005 (7)
C6	0.0147 (8)	0.0205 (9)	0.0220 (9)	−0.0043 (7)	0.0054 (7)	0.0003 (7)
C7	0.0199 (9)	0.0166 (9)	0.0288 (10)	−0.0027 (7)	0.0064 (8)	0.0033 (8)
C8	0.028 (1)	0.0254 (10)	0.0170 (8)	0.0006 (8)	0.0064 (7)	0.0000 (8)
N1'	0.0117 (7)	0.0138 (7)	0.0148 (7)	0.0000 (5)	0.0018 (5)	0.0018 (6)
C1'	0.0135 (8)	0.0234 (9)	0.0138 (8)	−0.0030 (7)	0.0005 (6)	0.0008 (7)
C2'	0.0153 (8)	0.0218 (9)	0.0142 (8)	−0.0034 (7)	0.0037 (6)	−0.0048 (7)
C3'	0.0111 (8)	0.0164 (8)	0.0213 (9)	−0.0035 (6)	0.0045 (7)	−0.0017 (7)
C4'	0.0130 (8)	0.0175 (8)	0.0153 (8)	−0.0009 (6)	0.0017 (6)	0.0013 (7)
C5'	0.0110 (7)	0.0151 (8)	0.0143 (8)	−0.0025 (6)	0.0021 (6)	0.0002 (7)
C6'	0.0125 (8)	0.0157 (8)	0.0213 (9)	0.0027 (6)	0.0006 (7)	0.0032 (7)
C7'	0.0164 (8)	0.0184 (9)	0.0246 (9)	0.0016 (7)	0.0044 (7)	0.0068 (7)
C8'	0.0194 (9)	0.0172 (9)	0.0266 (9)	−0.0003 (7)	0.0053 (7)	−0.0036 (8)
O1W	0.0309 (8)	0.0252 (8)	0.0342 (8)	−0.0016 (6)	0.0096 (6)	−0.0014 (7)

### Geometric parameters (Å, º)

**Table:**

N1—C1	1.348 (2)	N1'—C1'	1.344 (2)
N1—C5	1.359 (2)	N1'—C5'	1.358 (2)
N1—C6	1.491 (2)	N1'—C6'	1.492 (2)
C1—C2	1.372 (3)	C1'—C2'	1.375 (2)
C1—H1	0.9500	C1'—H1'	0.9500
C2—C3	1.390 (2)	C2'—C3'	1.389 (2)
C2—H2	0.9500	C2'—H2'	0.9500
C3—C4	1.393 (2)	C3'—C4'	1.396 (2)
C3—C8	1.494 (2)	C3'—C8'	1.495 (2)
C4—C5	1.374 (2)	C4'—C5'	1.377 (2)
C4—H4	0.9500	C4'—H4'	0.9500
C5—C5'	1.482 (2)	C6'—C7'	1.523 (2)
C6—C7	1.528 (3)	C6'—H6A'	0.9900
C6—H6A	0.9900	C6'—H6B'	0.9900
C6—H6B	0.9900	C7'—H7A'	0.9900
C7—C7'	1.523 (3)	C7'—H7B'	0.9900
C7—H7B	0.9900	C8'—H8A'	0.9800
C7—H7A	0.9900	C8'—H8B'	0.9800
C8—H8A	0.9800	C8'—H8C'	0.9800
C8—H8B	0.9800	O1W—H1W	0.809 (19)
C8—H8C	0.9800	O1W—H2W	0.807 (19)
			
C1—N1—C5	119.74 (15)	C1'—N1'—C6'	117.50 (14)
C1—N1—C6	117.59 (15)	C5'—N1'—C6'	122.62 (14)
C5—N1—C6	122.66 (14)	N1'—C1'—C2'	121.64 (16)
N1—C1—C2	121.59 (17)	N1'—C1'—H1'	119.2
N1—C1—H1	119.2	C2'—C1'—H1'	119.2
C2—C1—H1	119.2	C1'—C2'—C3'	120.09 (16)
C1—C2—C3	120.29 (16)	C1'—C2'—H2'	120.0
C1—C2—H2	119.9	C3'—C2'—H2'	120.0
C3—C2—H2	119.9	C2'—C3'—C4'	117.37 (16)
C2—C3—C4	116.94 (16)	C2'—C3'—C8'	121.61 (16)
C2—C3—C8	122.41 (16)	C4'—C3'—C8'	121.00 (16)
C4—C3—C8	120.65 (16)	C5'—C4'—C3'	120.79 (16)
C5—C4—C3	121.45 (16)	C5'—C4'—H4'	119.6
C5—C4—H4	119.3	C3'—C4'—H4'	119.6
C3—C4—H4	119.3	N1'—C5'—C4'	120.31 (15)
N1—C5—C4	119.97 (15)	N1'—C5'—C5	120.15 (15)
N1—C5—C5'	120.37 (15)	C4'—C5'—C5	119.46 (15)
C4—C5—C5'	119.58 (15)	N1'—C6'—C7'	111.60 (14)
N1—C6—C7	111.16 (14)	N1'—C6'—H6A'	109.3
N1—C6—H6A	109.4	C7'—C6'—H6A'	109.3
C7—C6—H6A	109.4	N1'—C6'—H6B'	109.3
N1—C6—H6B	109.4	C7'—C6'—H6B'	109.3
C7—C6—H6B	109.4	H6A'—C6'—H6B'	108.0
H6A—C6—H6B	108.0	C6'—C7'—C7	115.78 (15)
C7'—C7—C6	116.31 (15)	C6'—C7'—H7A'	108.3
C7'—C7—H7B	108.2	C7—C7'—H7A'	108.3
C6—C7—H7B	108.2	C6'—C7'—H7B'	108.3
C7'—C7—H7A	108.2	C7—C7'—H7B'	108.3
C6—C7—H7A	108.2	H7A'—C7'—H7B'	107.4
H7B—C7—H7A	107.4	C3'—C8'—H8A'	109.5
C3—C8—H8A	109.5	C3'—C8'—H8B'	109.5
C3—C8—H8B	109.5	H8A'—C8'—H8B'	109.5
H8A—C8—H8B	109.5	C3'—C8'—H8C'	109.5
C3—C8—H8C	109.5	H8A'—C8'—H8C'	109.5
H8A—C8—H8C	109.5	H8B'—C8'—H8C'	109.5
H8B—C8—H8C	109.5	H1W—O1W—H2W	104 (3)
C1'—N1'—C5'	119.79 (15)		
			
C5—N1—C1—C2	−1.2 (3)	C1'—C2'—C3'—C4'	−0.8 (2)
C6—N1—C1—C2	177.51 (16)	C1'—C2'—C3'—C8'	177.56 (16)
N1—C1—C2—C3	0.0 (3)	C2'—C3'—C4'—C5'	0.2 (2)
C1—C2—C3—C4	1.3 (2)	C8'—C3'—C4'—C5'	−178.18 (16)
C1—C2—C3—C8	−178.70 (17)	C1'—N1'—C5'—C4'	0.4 (2)
C2—C3—C4—C5	−1.5 (2)	C6'—N1'—C5'—C4'	−176.07 (15)
C8—C3—C4—C5	178.50 (16)	C1'—N1'—C5'—C5	177.22 (15)
C1—N1—C5—C4	1.0 (2)	C6'—N1'—C5'—C5	0.8 (2)
C6—N1—C5—C4	−177.65 (15)	C3'—C4'—C5'—N1'	0.0 (2)
C1—N1—C5—C5'	177.79 (15)	C3'—C4'—C5'—C5	−176.85 (15)
C6—N1—C5—C5'	−0.8 (2)	N1—C5—C5'—N1'	66.1 (2)
C3—C4—C5—N1	0.4 (3)	C4—C5—C5'—N1'	−117.12 (18)
C3—C4—C5—C5'	−176.45 (15)	N1—C5—C5'—C4'	−117.09 (18)
C1—N1—C6—C7	87.25 (19)	C4—C5—C5'—C4'	59.7 (2)
C5—N1—C6—C7	−94.09 (19)	C1'—N1'—C6'—C7'	88.23 (18)
N1—C6—C7—C7'	83.16 (19)	C5'—N1'—C6'—C7'	−95.23 (19)
C5'—N1'—C1'—C2'	−1.0 (2)	N1'—C6'—C7'—C7	82.48 (19)
C6'—N1'—C1'—C2'	175.61 (15)	C6—C7—C7'—C6'	−52.2 (2)
N1'—C1'—C2'—C3'	1.3 (3)		

### Hydrogen-bond geometry (Å, º)

**Table:**

*D*—H···*A*	*D*—H	H···*A*	*D*···*A*	*D*—H···*A*
C1—H1···Br2^i^	0.95	2.68	3.5929 (18)	161
C2—H2···Br1^ii^	0.95	2.86	3.7762 (18)	161
C7—H7*B*···Br1^i^	0.99	2.96	3.7700 (19)	139
C1′—H1′···Br2^iii^	0.95	2.64	3.5765 (17)	170
C2′—H2′···Br1^iv^	0.95	2.82	3.6285 (17)	143
C4′—H4′···Br1	0.95	2.74	3.6735 (17)	167
C7′—H7*B*′···Br1^v^	0.99	3.04	3.6581 (18)	122
O1*W*—H1*W*···Br1	0.81 (2)	2.58 (2)	3.3856 (15)	177 (3)
O1*W*—H2*W*···Br2	0.81 (2)	2.56 (2)	3.3670 (15)	175 (3)

Symmetry codes: (i) −*x*+1/2, *y*−1/2, −*z*+1/2; (ii) −*x*+1, −*y*+1, −*z*; (iii) −*x*+1, −*y*+1, −*z*+1; (iv) *x*+1/2, −*y*+3/2, *z*+1/2; (v) −*x*+3/2, *y*−1/2, −*z*+1/2.
