# Supplementary figures and images for: 4,15-Dimethyl-7,12-diazo­niatri­cyclo­[10.4.0.02,7]hexa­deca-1(12),2,4,6,13,15-hexa­ene dibromide monohydrate
Source: Acta Crystallogr E Crystallogr Commun. 2020 Aug 18;76(Pt 9):1467–71. doi: 10.1107/S2056989020011147 (PMC7472763; doi:10.1107/S2056989020011147)

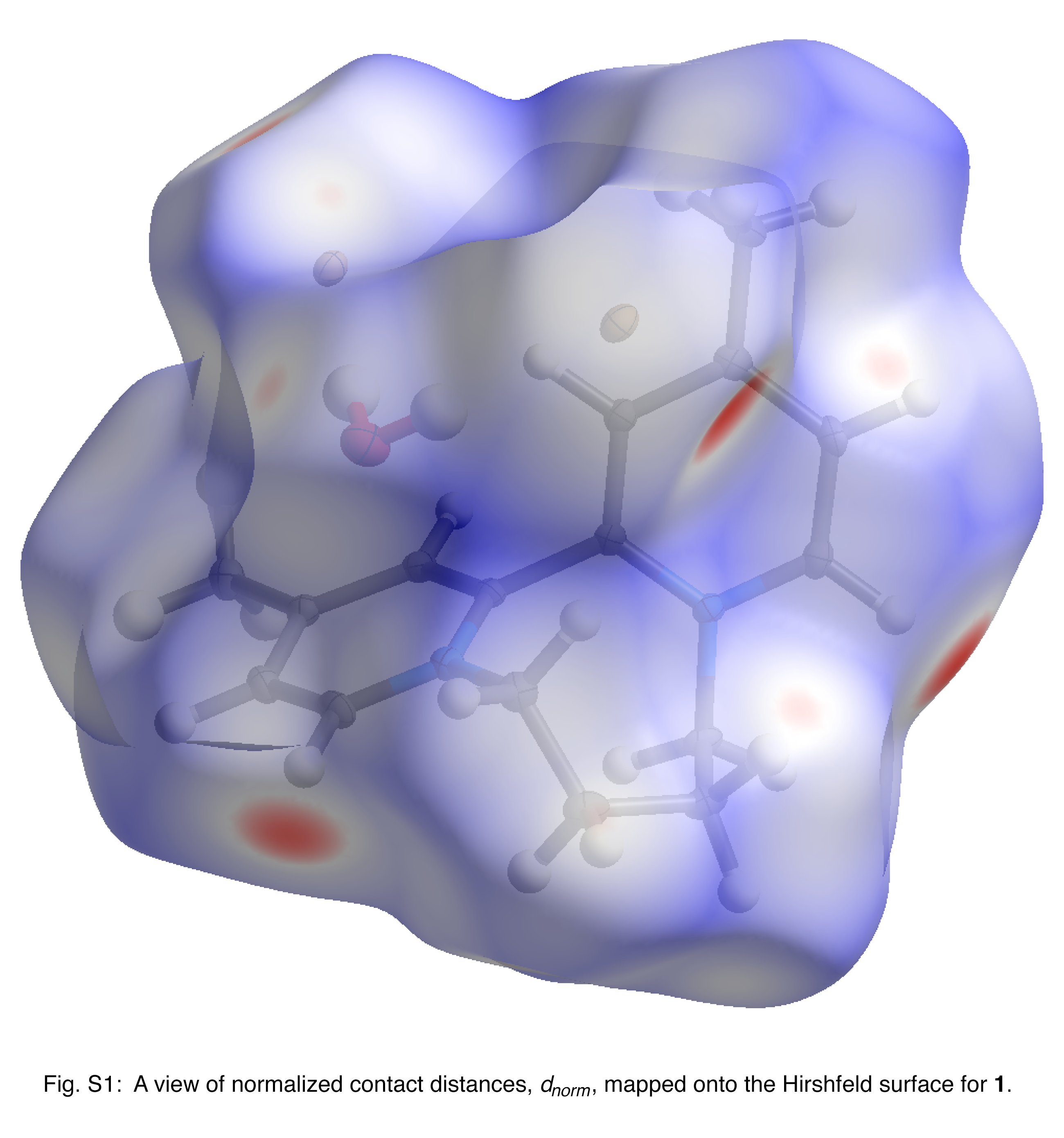

Supplement: Supplementary file 3 [file e-76-01467-sup3.tif]

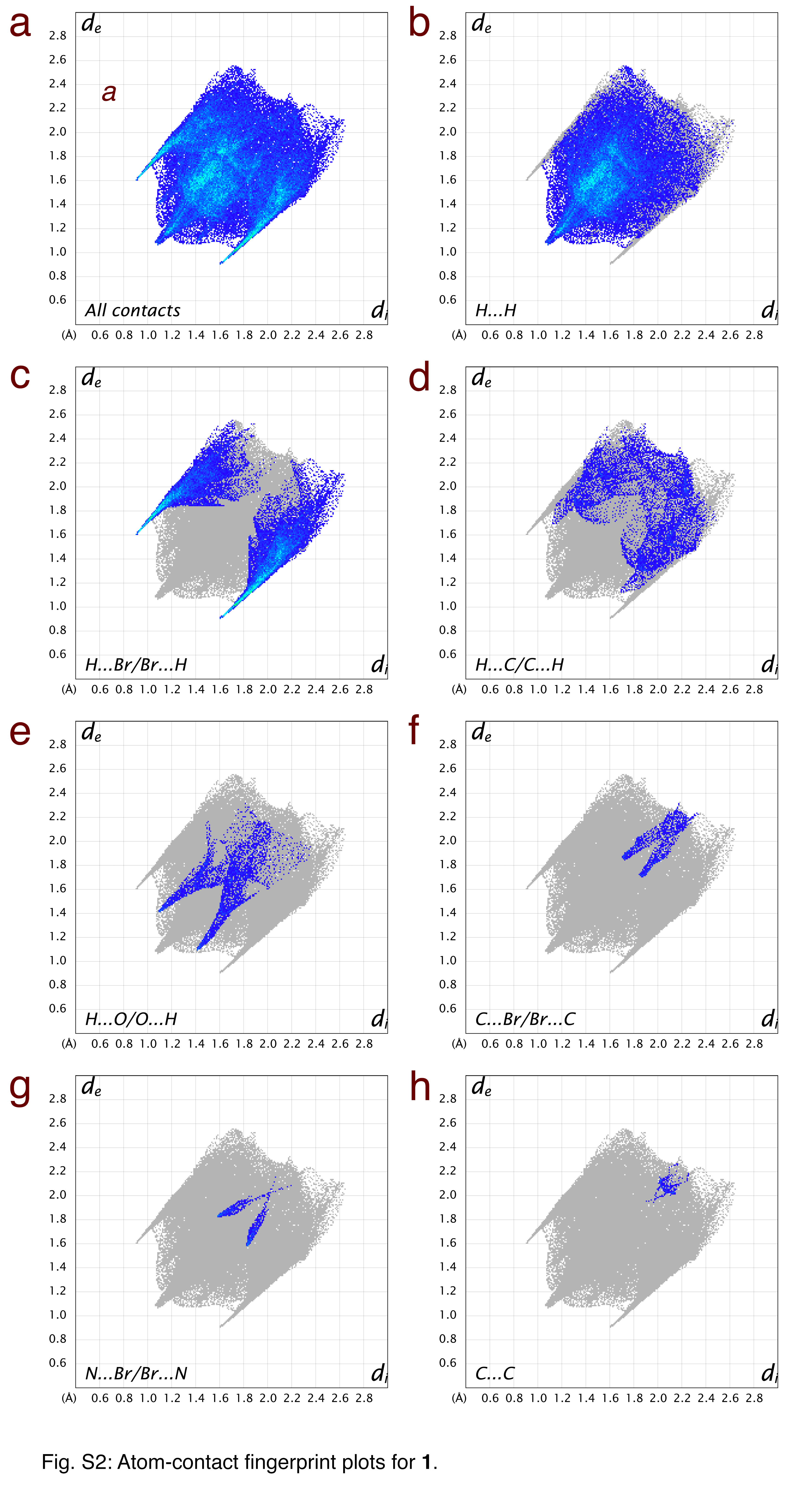

Supplement: Supplementary file 4 [file e-76-01467-sup4.tif]
